# Soluble fms‐like tyrosine kinase‐1 and placental growth factor as predictors of adverse maternal events in women with a confirmed diagnosis of preeclampsia

**DOI:** 10.1002/pmf2.70293

**Published:** 2026-04-23

**Authors:** Niclas Carlberg, Camilla Hesse, Sven‐Egron Thörn, Tor Damén, Henrik Imberg, Nicole Wallin, Malin Andersson, Verena Sengpiel, Lina Bergman

**Affiliations:** ^1^ Department of Anesthesiology and Intensive Care Medicine Sahlgrenska University Hospital Östra Gothenburg Sweden; ^2^ Department of Obstetrics and Gynecology Institute of Clinical Sciences Sahlgrenska Academy University of Gothenburg Gothenburg Sweden; ^3^ Department of Laboratory Medicine Institute of Biomedicine Gothenburg University Gothenburg Sweden; ^4^ Department of Anesthesiology and Intensive Care Medicine Institute of Clinical Sciences at the Sahlgrenska Academy University of Gothenburg Gothenburg Sweden; ^5^ Section of Cardiothoracic Anesthesia and Intensive Care Sahlgrenska University Hospital Gothenburg Sweden; ^6^ Statistiska Konsultgruppen Gothenburg Sweden; ^7^ Department of Obstetrics and Gynecology Sahlgrenska University Hospital Gothenburg Sweden; ^8^ Department of Obstetrics and Gynecology Stellenbosch University Cape Town South Africa; ^9^ Department of Women's and Children's Health Uppsala University Uppsala Sweden

**Keywords:** adverse maternal events, angiogenic markers, hypertensive disorders of pregnancy, placental growth factor (PlGF), preeclampsia, pregnancy‐related complications, soluble fms‐like tyrosinekinase‐1 (sFlt‐1)

## Abstract

**Objective:**

Soluble fms‐like tyrosine kinase‐1 (sFlt‐1) and placental growth factor (PlGF) have been identified as predictors for preeclampsia and time to birth in women with suspected preeclampsia. This study aims to investigate these markers as predictors of adverse maternal events in women with confirmed preeclampsia.

**Study design:**

This was a prospective observational cohort study. Pregnant women admitted to hospitals due to preeclampsia were included from May 2019 to May 2024.

**Main outcome measures:**

The primary outcome was a composite of adverse maternal events (maternal mortality, eclampsia, stroke, cortical blindness, retinal detachment, pulmonary edema, acute kidney injury, liver capsule hematoma or rupture, placental abruption, postpartum hemorrhage, elevated liver enzymes, thrombocytopenia, admission to intensive care unit (ICU) required, and intubation and mechanical ventilation), with the individual components as secondary outcomes. Kaplan–Meier and receiver operating characteristic curves were constructed for sFlt‐1, PlGF, and the sFlt‐1/PlGF ratio. Area under the curve (AUC), sensitivity, specificity, positive and negative predicted value, and likelihood ratio were calculated.

**Results:**

A total of 307 women were enrolled, of which 36.5% gave birth preterm. In total, 129 women developed 166 adverse maternal events. The most common adverse maternal events were postpartum hemorrhage (*n* = 82, 26.7%), elevated liver enzymes (*n* = 34, 11.1%), acute kidney injury (*n* = 24, 7.8%), and low platelet count (*n* = 18, 5.9%). sFlt‐1 showed the best predictive performance, with an area under the receiver operating characteristic curve for the composite outcome of 0.59 (95% confidence interval [CI] 0.52−0.65), corresponding to a sensitivity of 67% and a specificity of 50%. PlGF and the sFlt‐1/PlGF ratio did not predict the composite outcome. sFlt‐1 showed a modest predictive performance for thrombocytopenia (AUC, 0.68; 95% CI, 0.56–0.81), elevated liver enzymes (AUC, 0.68; 95% CI, 0.59–0.78), and renal impairment (AUC, 0.72; 95% CI, 0.60–0.83). Results remained similar in subgroup analyses of early‐ and late‐onset, and preterm and term preeclampsia.

**Conclusion:**

SFlt‐1 and PlGF show limited value in predicting adverse maternal events in women with preeclampsia in a high‐income country setting, irrespective of early or late disease. sFlt‐1 may be useful for predicting the individual outcomes of thrombocytopenia, elevated liver enzymes, and acute kidney injury.

## 1 INTRODUCTION

Preeclampsia is a pregnancy‐induced disorder and a common cause of direct pregnancy‐related morbidity and mortality. Diagnosis and complications of this disease are challenging to predict. The angiogenic markers soluble fms‐like tyrosine kinase (sFlt‐1), placental growth factor (PlGF), and the sFlt‐1/PlGF ratio are currently being investigated for their ability to predict the onset of preeclampsia, time to delivery, and adverse maternal events in pregnant women presenting with suspected preeclampsia [1, 2, 3].

Few previous studies have investigated the predictive value of angiogenic markers for adverse maternal events in women with a confirmed diagnosis of preeclampsia. A recent systematic review on the prediction of adverse maternal and perinatal complications in all hypertensive disorders of pregnancy showed that sFlt‐1 and PlGF might be useful in predicting certain outcomes. These included intensive care unit (ICU) admission, elevated liver enzymes, low platelets, hemolysis, elevated liver enzymes, and low platelet count (HELLP) syndrome, and placental abruption. However, studies investigating angiogenic markers were few and small with inconsistent results. In addition, they used different outcomes, precluding pooling of results across studies [4]. In 2020, a consensus for core maternal and fetal outcomes in preeclampsia was developed to standardize outcomes used in preeclampsia research and facilitate comparisons between studies [5]. We aimed to investigate whether angiogenic markers can predict adverse maternal events according to this core set of outcomes in women with a confirmed diagnosis of preeclampsia.

## 2 MATERIALS AND METHODS

This is a prospective, observational cohort study, based on the Gothenburg Preeclampsia Obstetric adverse events study (GoPROVE) [6]. Ethical approval was obtained on December 28, 2018, from the Ethical Review Board Gothenburg (No. 955–18), with amendments approved on April 28, 2023, by Ethix, the Swedish Ethical Review Authority (No. 2023‐02‐271‐02). The GoPROVE Database and Biobank is registered at ISCRTN (13060768) 2020‐07‐27. All participants gave informed consent before data collection. The study was conducted in accordance with the Declaration of Helsinki.

### 2.1 Population

Participants were recruited from May 2019 to May 2024 at Sahlgrenska University Hospital, Sweden, following hospital admission due to a diagnosis of preeclampsia. Sahlgrenska University Hospital is a tertiary center for obstetric care with approximately 10 000 deliveries per year. In Sweden, angiogenic markers are currently not being used for the prediction or diagnosis of preeclampsia, and the antenatal care program is provided free of charge and has almost 100% adherence. Admission to the hospital follows national guidelines for preeclampsia care. Induction of labor is recommended within 48 h when preeclampsia is diagnosed after 37+0 gestational weeks. If preeclampsia is diagnosed before 37+0 gestational weeks and there are no signs of maternal or fetal deterioration, expectant management is offered. Surveillance includes at a minimum twice weekly blood pressure and biochemical assessment and bi‐weekly growth scans with fetal Doppler assessment as needed. For inpatient care, daily assessment of blood pressure and CTG are normally indicated. Indication for delivery includes signs of maternal deterioration or fetal compromise similar to international guidelines.Preeclampsia was diagnosed as new‐onset hypertension (> 140/90 mmHg on two occasions at least 15 min apart) after 20 gestational weeks and excessive proteinuria, defined as a urine albumin/creatinine ratio ≥8 mg/mmol, urine protein/creatinine ratio ≥30, or urine dipstick ≥2+. In cases where neither a protein‐to‐creatinine ratio nor an albumin‐to‐creatinine ratio was analyzed, a urine dipstick result of ≥2+ was accepted as evidence of significant proteinuria. Adverse maternal events are defined in the Outcomes section. Superimposed preeclampsia was defined as new‐onset proteinuria after 20 gestational weeks in women with chronic hypertension. Exclusion criteria included preexisting cardiac, neurologic, or renal disease. Only participants with preeclampsia without any adverse maternal events at the time of inclusion were eligible for this study.

### 2.2 Exposures

Exposures were circulating concentrations of sFlt‐1 (pg/mL) and PlGF (pg/mL), and the sFlt‐1/PlGF ratio, at inclusion.

### 2.3 Outcomes

The primary outcome was a composite of maternal adverse events, as recommended in a Delphi consensus by Duffy et al. [5]. These included maternal mortality, eclampsia, stroke, cortical blindness, retinal detachment, pulmonary edema, acute kidney injury, liver capsule hematoma or rupture, placental abruption, postpartum hemorrhage, raised liver enzymes, low platelets, admission to ICU required, and intubation and mechanical ventilation (not for childbirth), whose definitions are listed in Table S1. All individual outcomes with ≥10 events in the study population were classified as secondary individual outcomes, in addition to the primary composite outcome. Furthermore, an ad hoc secondary analysis was performed using the composite outcome excluding postpartum hemorrhage, given that sFlt‐1 and PlGF might not be biologically plausible predictors of postpartum hemorrhage.

### 2.4 Variables

Clinical and laboratory data on background characteristics and outcomes were retrieved from the participant's medical charts or through patient questionnaires. Data were stored in an electronic case report form hosted by Omda. All data were double‐checked for accuracy.

### 2.5 Sample collection

Venipuncture was performed as close to the time of inclusion as possible, typically shortly after the diagnosis of preeclampsia. Blood was collected in serum tubes without gel. Samples were centrifuged at 2000 g within 24 h before being aliquoted and stored at −80°C using the hospital integrated biobank, Biobank Väst.

### 2.6 Biomarker analysis

Serum concentrations of sFlt‐1 and PlGF were analyzed using the automatized platform DELFIA Xpress 1‐2‐3 (Perkin‐Elmer) and the assays (Revvity Inc, Wallac Oy) at iLab Medical AB [7]. The coefficients of variation were 2.3% and 3.8% among the standards and controls for sFlt‐1 and PlGF, respectively. Laboratory personnel were blinded to the study groups.

### 2.7 Statistics

Descriptive statistics were reported as mean (standard deviation, SD) or median (interquartile range, IQR) for numeric variables, and as counts (percentages) for categorical variables. All biomarkers were log‐transformed to account for skewed distributions of data, adjusted for gestational age using linear (sFlt‐1) or quadratic (PlGF and the sFlt‐1/PlGF ratio) regression models, and expressed as multiple of medians (MoM), defined as the residual from the respective regression models. Kaplan–Meier survival curves of time to first event were presented for participants below or above the gestational age–adjusted median of each biomarker. Differences in time to event were assessed using the log‐rank test.

Predictive accuracy was assessed using receiver operating characteristic (ROC) curves, with area under the curve (AUC) and 95% confidence intervals (CIs). Optimal cutoffs were determined using Youden's J‐index and the minimal distance to (0, 1). These analyses were performed for the composite outcome and for all individual outcomes with ≥10 events in the study population. Subgroup analyses were performed for participants with early‐onset (< 34+0 gestational weeks) and late‐onset (≥34+0 gestational weeks), and preterm (< 37+0 gestational weeks) and term (≥37+0 gestational weeks) preeclampsia. Subgroup analyses were performed to aid comparison with previous studies and were unadjusted for gestational age.

For the analyses with DELFIA Xpress, there were no previously described optimal cutoff values for any of the biomarkers for adverse maternal events, but there were some recommended cutoffs for ruling in and ruling out preeclampsia in women with suspected preeclampsia. The rule‐in criteria included PlGF < 50 pg/mL or sFlt‐1/PlGF > 70 or > 90 for women with gestational age before or after 34+0 weeks. The rule‐out criteria included PlGF > 150 pg/mL or sFlt‐1/PlGF < 50 [7]. Sensitivity, specificity, positive and negative predictive value, and positive and negative likelihood ratio were calculated for the composite outcome using these cutoffs. Statistical analyses were performed using IBM SPSS Statistics for Windows, Version 28.0 (IBM Corp.), and R software version 4.3.0 (R Foundation for Statistical Computing) and epiR package version 2.0.82.

## 3 RESULTS

**FIGURE 1:**
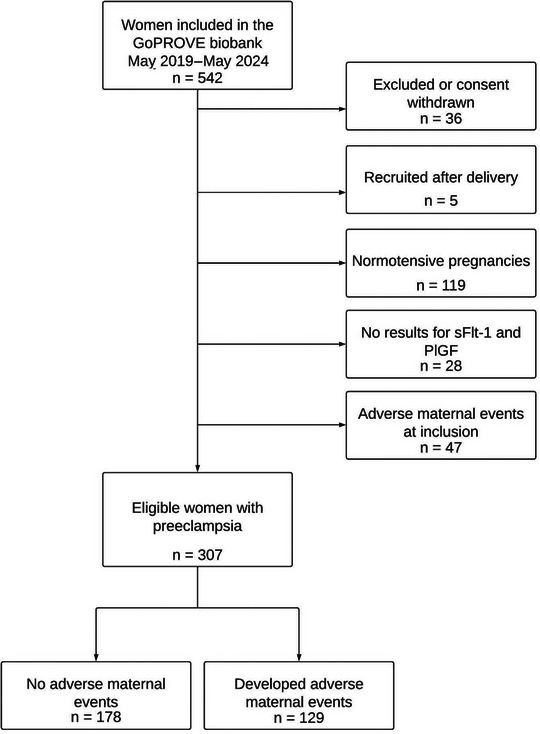
Flowchart of participant inclusion and study population. GoPROVE, Gothenburg Preeclampsia Obstetric adverse events study; PlGF, placental growth factor; sFlt‐1, soluble FMS‐like tyrosine kinase‐1.

A total of 307 participants with preeclampsia without adverse maternal events at the time of inclusion were enrolled in the study. A flow chart of the study population is presented in Figure 1.

The participants included in this study were on average 31 years old (SD 5.0), and 239 (77.9%) were nulliparous. At inclusion, the median gestational age was 37+3 (IQR 34+4–39+0) weeks. Participants who developed adverse maternal events were more often nulliparous (82.9% vs. 74.2%), less often delivered preterm (31.8% vs. 39.9%), and more often gave birth vaginally (59.7% vs. 50.6%), compared with participants who did not develop adverse maternal events (Table 1).

**TABLE 1:** Maternal and fetal characteristics at enrollment and birth in participants with preeclampsia, stratified by the presence or absence of adverse maternal events.

		Adverse maternal events	
Maternal or fetal characteristics	All participants (*n* = 307)	Yes (*n* = 129)	No (*n* = 178)
Age (years)	31 (5.0)	31 (4.3)	32 (5.3)
Nulliparous	239 (77.9%)	107 (82.9%)	132 (74.2%)
Multipara	5 (1.6%)	3 (2.3%)	≤3 (≤1.7%)
Body mass index (kg/m^2^)	26.8 (5.7)	26.5 (6.0)	27.0 (5.4)
Diabetes mellitus type I	3 (1.0%)	≤3	≤3
Diabetes mellitus type II	≤3	≤3	≤3
Gestational diabetes	11 (3.6%)	≤3 (≤2.3%)	9 (5.1%)
Chronic hypertension	14 (4.6%)	4 (3.1%)	10 (5.6%)
Renal disease	≤3	≤3	≤3
Smoking at any time during pregnancy	12 (3.9%)	≤3 (≤2.3%)	9 (5.1%)
Alcohol use at any time during pregnancy	≤3	≤3	≤3
At inclusion			
Gestational age at blood sampling (weeks+days)	37+3 (34+4–39+0)	37+5 (35+1–39+2)	37+2 (34+2–38+5)
Systolic blood pressure (mmHg)	143 (13)	142 (13)	144 (14)
Diastolic blood pressure (mmHg)	90 (9)	89 (8)	90 (10)
Hemoglobin (g/dL)	12.0 (1.1)	12.0 (1.1)	12.0 (1.0)
Platelet count (×10^9^)	211 (57)	204 (58)	216 (57)
Creatinine (µmol/L)	57 (11)	59 (12)	55 (11)
Alanine aminotransferase (U/L)	17.4 (13.8)	19.2 (15.6)	16.2 (12)
Duration of antihypertensive treatment before delivery (days)	4 (1–13)	4 (1–9)	5 (2–18)
At delivery			
Gestational age at birth (weeks+days)	37+4 (35+4–39+1)	38+0 (36+0–39+4)	37+3 (35+1–38+6)
Delivery before 37+0 weeks’ gestational age	112 (36.5%)	41 (31.8%)	71 (39.9%)
Days from diagnosis of preeclampsia to delivery	4 (2–9)	4 (2–8)	4 (2–10)
Liveborn	305 (99.3%)	128 (99.2%)	177 (99.4%)
Birthweight (g)	2765 (928)	2854 (921)	2700 (930)
Birthweight centile	34.0 (32.3)	36.0 (32.4)	32.5 (32.3)
Vaginal delivery	167 (54.4%)	77 (59.7%)	90 (50.6%)
Cesarean section	140 (45.6%)	52 (40.3%)	88 (49.4%)
Severe hypertension	214 (69.7%)	91 (70.5%)	123 (69.1%)

*Note*: Numeric variables are presented as mean (standard deviation) or median (interquartile range), as appropriate. Categorical variables are presented as counts and percentages.

Baseline plasma concentrations of angiogenic markers with and without adjustments for gestational age are presented in Table 2 (calculations are provided in Table S2). Differences in the median concentrations of sFlt‐1 and PlGF between groups were minor. Adverse maternal events are presented in Table 3. In total, 129 participants had 166 adverse maternal events. The mean number of events per participant was 1.3, and the mean time from blood sampling to event was 3.2 days. The most common adverse maternal event was postpartum bleeding, followed by elevated liver enzymes, acute kidney injury, low platelets, placental abruption, admission to ICU, pulmonary edema, and eclampsia. There was no maternal death, cerebrovascular accident, cortical blindness, retinal detachment, hepatic rupture, or respiratory failure.

**TABLE 2:** Baseline angiogenic markers in participants with preeclampsia, stratified by the presence or absence of adverse maternal events.

Angiogenic marker	Preeclampsia (*n* = 307)	Adverse maternal events	
		Yes (*n* = 129)	No (*n* = 178)
sFlt‐1 (pg/mL)	4092 (2802–5947)	4343 (3212–6187)	3989 (2565–5656)
PlGF (pg/mL)	49.6 (32.6–74.3)	50.3 (34.0–78.5)	48.4 (31.3–71.3)
sFlt‐1/PlGF ratio	80 (49–146)	92 (50–145)	77 (48–146)
GA‐adjusted sFlt‐1 (log MoM)	0.07 (−0.33 to 0.35)	0.14 (−0.21 to 0.45)	0.01 (−0.41 to 0.29)
GA‐adjusted PlGF (log MoM)	−0.02 (−0.38 to 0.34)	−0.06 (−0.35 to 0.46)	−0.01 (−0.39 to 0.29)
GA‐adjusted sFlt‐1/PlGF ratio (log MoM)	0.05 (−0.47 to 0.57)	0.17 (−0.47 to 0.63)	0.02 (−0.51 to 0.51)

*Note*: Values are presented as median (interquartile range).

Abbreviations: GA, gestational age; MoM, multiples of median; PlGF, placental growth factor; sFlt‐1, soluble fms‐like tyrosine kinase.

**TABLE 3:** Frequency of adverse maternal events.

Maternal adverse event	*n*	Percentage of participants	Percentage of events
All maternal adverse events	166	42.0%	100%
Postpartum hemorrhage	82	26.7%	49.4%
Elevated liver enzymes	34	11.1%	20.5%
Acute kidney injury	24	7.8%	14.5%
Low platelet count	18	5.9%	10.8%
Placental abruption	5	1.6%	3.0%
Admission to ICU	≤3	≤1.0%	≤1.8%
Eclampsia	≤3	≤1.0%	≤1.8%
Pulmonary edema	≤3	≤1.0%	≤1.8%
Cerebrovascular accident	0	0.0%	0.0%
Cortical blindness	0	0.0%	0.0%
Hepatic rupture	0	0.0%	0.0%
Maternal death	0	0.0%	0.0%
Respiratory failure	0	0.0%	0.0%
Retinal detachment	0	0.0%	0.0%
HELLP syndrome	4	1.3%	2.4%

*Note*: Mean time (SD) to first event: 3.2 (4.2) days. Mean number (SD) of complications per affected woman: 1.3 (0.6).

Abbreviations: HELLP, hemolysis, elevated liver enzymes, and low platelet count; ICU, intensive care unit.

### 3.1 sFlt‐1 and PlGF as predictors for the composite adverse maternal outcome

**FIGURE 2:**
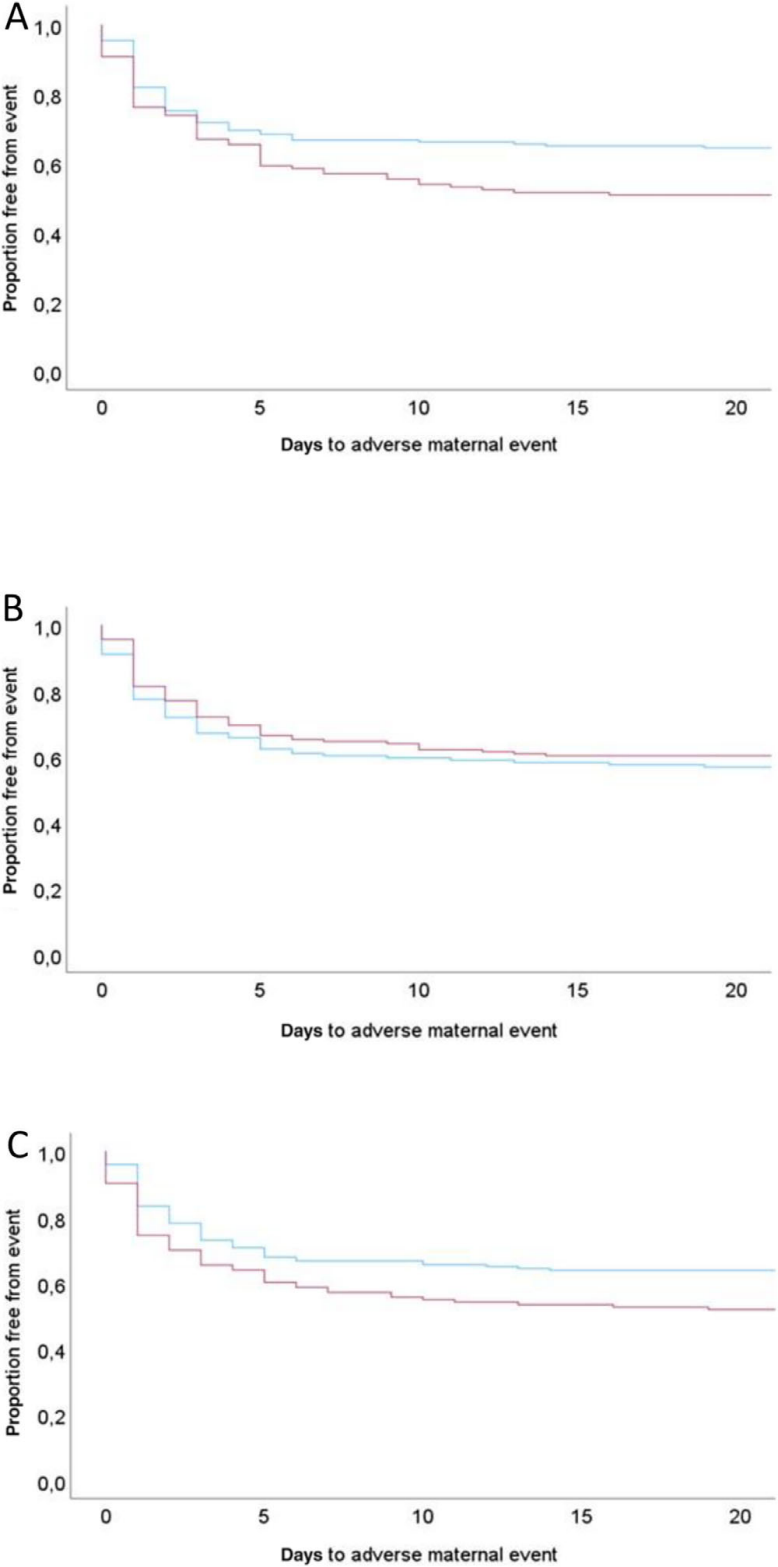
Kaplan–Meier curves for time to first adverse maternal event, stratified by biomarker levels above (red line) or below (blue line) the gestational age–adjusted multiple of median, based on sFlt‐1 (A), PlGF (B), and the sFlt‐1/PlGF ratio (C). *p* = 0.021 (sFlt‐1); *p* = 0.445 (PlGF); *p* = 0.028 (sFlt‐1/PlGF). Blue line: participants with plasma concentrations of biomarkers below the median. Red line: participants with plasma concentrations above the median. *p*‐values are calculated with the log rank (Mantel–Cox) test. sFlt‐1, soluble fms‐like tyrosine kinase; PlGF, placental growth factor.

**FIGURE 3:**
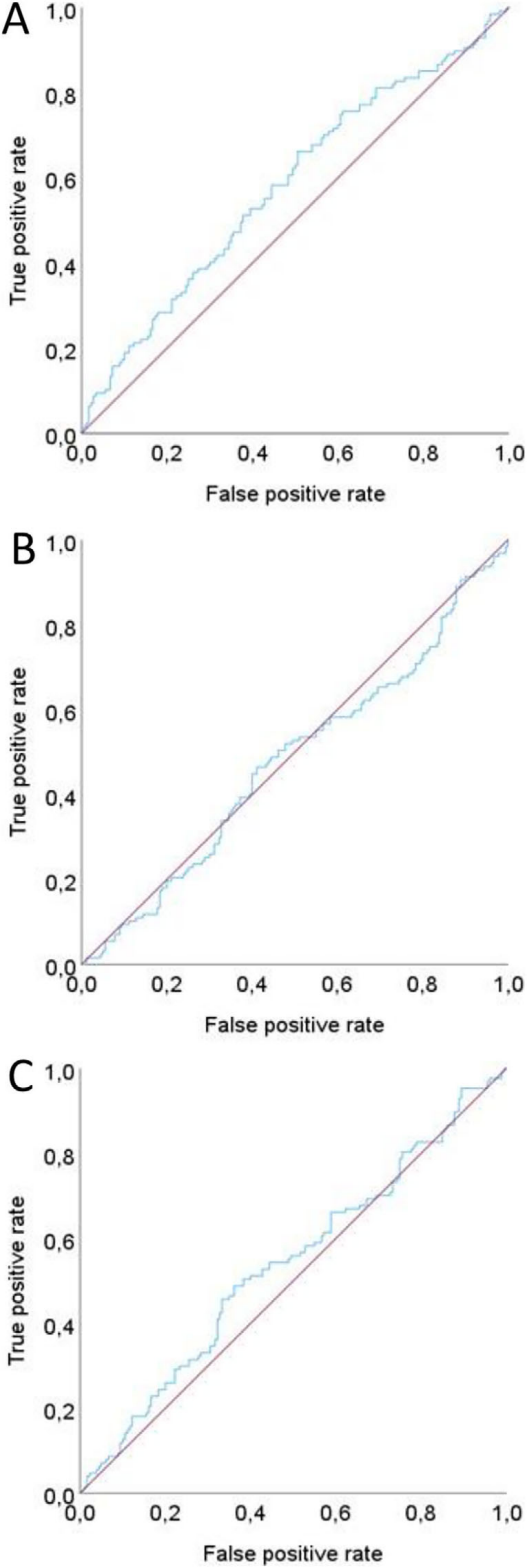
ROC curves for circulating concentrations of sFlt‐1, PlGF, and sFlt‐1/PlGF in predicting any adverse maternal event, based on the core outcome set for preeclampsia research according to Duffy et al. [5]. (A) sFlt‐1: AUC, 0.59 (95% CI, 0.52–0.65); (B) PlGF: AUC, 0.48 (95% CI, 0.42–0.55); (C) sFlt‐1/PlGF: AUC, 0.54 (95% CI, 0.48–0.61). AUC, area under the curve; PlGF, placental growth factor; ROC, receiver operating characteristics; sFlt‐1, soluble fms‐like tyrosine kinase.

In the time‐to‐event analysis, participants with sFlt‐1 and sFlt‐1/PlGF above median more often experienced the composite outcome (Figure 2). The AUC for sFlt‐1 predicting the composite outcome was 0.59 (95% CI, 0.52−0.65). The optimal cutoffs according to Youden's J‐index and minimal distance were 2% below and 8% above the gestational age–specific median for sFlt‐1, respectively, translating to sensitivities of 67% and 59%, with specificities of 50% and 66%. The AUCs for PlGF and the sFlt‐1/PlGF ratio predicting the composite outcome were 0.48 (95% CI, 0.42−0.55) and 0.54 (95% CI, 0.48−0.61), respectively (Figure 3 and Table 4).

**TABLE 4:** Optimal gestational age–adjusted cutoffs for angiogenic biomarkers predicting adverse maternal events, and their corresponding sensitivities, specificities, positive and negative predictive values, and positive and negative likelihood ratios in participants with preeclampsia.

Biomarker/criterion	Cutoff	Sensitivity	Specificity	PPV	NPV	LR+	LR−
sFlt‐1							
Youden's J‐index	−0.025	0.67 (0.58–0.75)	0.50 (0.42–0.58)	0.49 (0.42–0.57)	0.67 (0.59–0.75)	1.33 (1.10–1.61)	0.67 (0.50–0.89)
Minimal distance	0.073	0.59 (0.50–0.67)	0.56 (0.49–0.64)	0.49 (0.41–0.58)	0.65 (0.57–0.73)	1.34 (1.08–1.68)	0.73 (0.57–0.93)
PlGF	−0.114	0.47 (0.38–0.55)	0.59 (0.51–0.66)	0.45 (0.36–0.54)	0.60 (0.53–0.68)	1.13 (0.88–1.46)	0.91 (0.74–1.11)
sFlt‐1/PlGF	0.205	0.49 (0.40–0.58)	0.64 (0.57–0.71)	0.50 (0.41–0.59)	0.63 (0.56–0.70)	1.36 (1.04–1.77)	0.80 (0.65–0.98)

*Note*: Sensitivity, specificity, PPV, NPV, LR+, and LR− are given as numbers (95% CI). The outcome was any adverse maternal event as defined by the core outcome set for preeclampsia research by Duffy et al. [5]. Cutoff values are based on log‐transformed, gestational age–adjusted multiples of median (log MoM). If not specified, optimal cutoff values were equal when calculated with Youden's J‐index and minimal distance. Youden's J‐index: maximizing the sum of the sensitivity and specificity. Minimal distance: minimizing the distance from the ROC curve to the optimal point (0, 1). sFlt‐1 MoM = sFlt‐1/exp(9.778 − 0.00585 * GA). PlFG MoM = PlGF exp(−2.692 + 0.44 * GA − 0.0000715 * GA^2^). sFlt‐1/PlGF MoM = sFlt‐1/PlGF ratio/exp(9.854 − 0.02763 * GA + 0.0000241 * GA^2^).

Abbreviations: GA, gestational age; LR−, negative likelihood ratio; LR+, positive likelihood ratio; MoM, multiple of median; NPV, negative predictive value; PlFG, placental growth factor; PPV, positive predictive value; ROC, receiver operating characteristic; sFlt‐1, soluble fms‐like tyrosine kinase 1.

For the prediction of adverse maternal events in established disease, the recommended cutoffs from the manufacturer of DELFIA Xpress and the assays showed poor predictive value (Table S3).

Two neonates were stillborn. In a sensitivity analysis, excluding these two cases, the results remained similar (Table S4).

### 3.2 sFlt‐1 and PlGF as predictors for individual adverse maternal events

**FIGURE 4:**
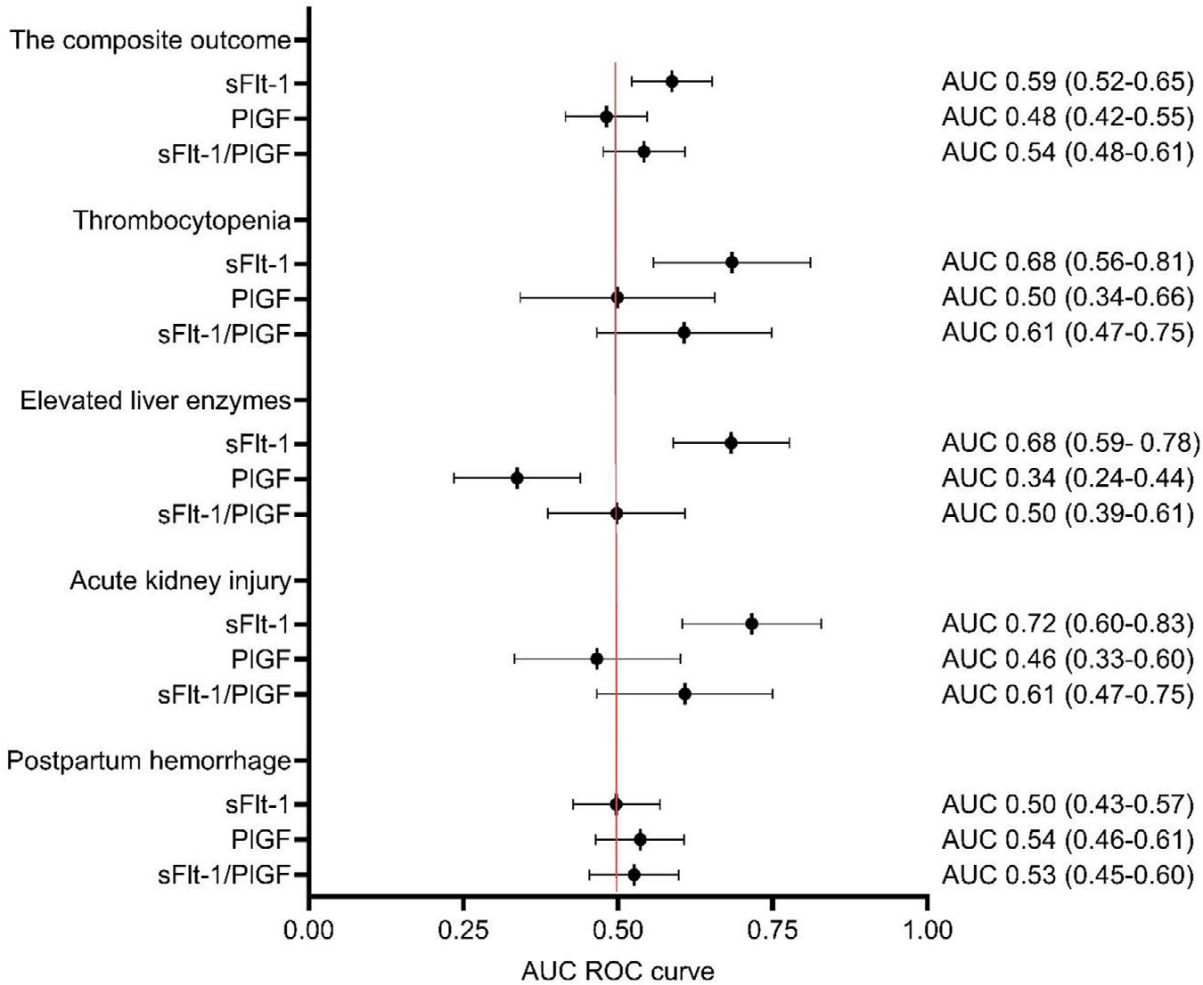
Forest plot of ROC AUC for predicting maternal adverse events, including the Delphi consensus composite outcome [5] and individual outcomes. Points represent ROC AUC, and error bars indicate 95% CI. The red vertical line denotes an ROC AUC of 0.5, indicating no discriminatory ability. CI, confidence intervals; PlGF, placental growth factor; ROC AUC, area under the receiver operating curve; sFlt‐1, soluble fms‐like tyrosine kinase.

sFlt‐1 was found to be associated with thrombocytopenia (AUC, 0.68; 95% CI, 0.56–0.81), elevated liver enzymes (AUC, 0.68; 95% CI, 0.59–0.78), and acute kidney injury (AUC, 0.72; 95% CI, 0.60–0.83), but not with postpartum hemorrhage. Lower PlGF levels showed an inverse association with elevated liver enzymes (AUC, 0.34; 95% CI, 0.24−0.44), indicating that higher PlGF concentrations were associated with increased risk. The sFlt‐1/PlGF ratio was not predictive of any adverse maternal event (Figure 4 and Table S5).

### 3.3 sFlt‐1 and PlGF as predictors of maternal adverse events in early‐onset and late‐onset preeclampsia

There were 63 and 244 participants categorized as having early‐ versus late‐onset preeclampsia (blood samples at diagnosis taken < 34 weeks’ or ≥34 weeks’ gestational age), respectively (Table S6). For early‐onset preeclampsia, sFlt‐1 was associated with the composite outcome with an AUC of 0.65 (95% CI, 0.50–0.79). Among individual adverse maternal events, sFlt‐1 and sFlt‐1/PlGF predicted acute kidney injury, with respective AUCs of 0.75 (95% CI, 0.54–0.97) and 0.76 (95% CI, 0.61–0.91) (Figure S1).

For late‐onset preeclampsia, none of the markers predicted the composite outcome. Among individual adverse maternal events, sFlt‐1 predicted thrombocytopenia (AUC, 0.69; 95% CI, 0.57–0.81), elevated liver enzymes (AUC, 0.70; 95% CI, 0.60–0.79), and acute kidney injury (AUC, 0.71; 95% CI, 0.59–0.83). Higher PlGF concentrations were associated with increased risk for elevated liver enzymes (Figure S2).

### 3.4 sFlt‐1 and PlGF as predictors for maternal adverse events in preterm and term preeclampsia

There were 128 and 179 participants categorized as having preterm versus term preeclampsia (blood samples at diagnosis taken < 37 weeks’ or ≥37 weeks’ gestational age), respectively (Table S6). For preterm preeclampsia, none of the markers predicted the composite outcome. Among individual adverse maternal events, sFlt‐1 and sFlt‐1/PlGF predicted thrombocytopenia, with AUCs of 0.75 (95% CI, 0.54–0.96) and 0.72 (95% CI, 0.57–0.87), respectively, and acute kidney injury, with respective AUCs of 0.70 (95% CI, 0.55–0.86) and 0.69 (95% CI, 0.55–0.83) (Figure S3).

For term preeclampsia, none of the angiogenic markers predicted the composite outcome. Among individual adverse maternal events, sFlt‐1 was predictive of thrombocytopenia (AUC, 0.66; 95% CI, 0.53–0.78), elevated liver enzymes (AUC, 0.76; 95% CI, 0.64–0.87), and acute kidney injury (AUC, 0.71; 95% CI, 0.55–0.87) (Figure S4).

### 3.5 sFlt‐1 and PlGF as predictors of the composite outcome excluding postpartum hemorrhage

sFlt‐1 demonstrated poor‐to‐moderate predictive performance for the composite outcome without postpartum hemorrhage in all women (AUC, 0.69; 95% CI, 0.62–0.77), in women with early‐onset preeclampsia (AUC, 0.66; 95% CI, 0.51–0.82), in women with late‐onset preeclampsia (AUC, 0.71; 95% CI, 0.63–0.79), in women with preterm preeclampsia (AUC, 0.63; 95% CI, 0.52–0.74), and in women with term preeclampsia (AUC, 0.77; 95% CI, 0.68–0.85). PlGF alone was not predictive in any subgroup. The sFlt‑1/PlGF ratio was predictive only in women with term preeclampsia (AUC, 0.63; 95% CI, 0.52–0.74) (Figure S5). The optimal cutoffs for sFlt‐1, PlGF, and the sFlt‐1/PlGF ratio, derived using Youden's J‐index and minimal distance, demonstrated generally poor predictive performance for the composite outcome excluding postpartum hemorrhage (Table S7).

## 4 DISCUSSION

In a Swedish high‐income setting, the angiogenic markers sFlt‐1, PlGF, and the sFlt‐1/PlGF ratio showed poor predictive performance for a composite of adverse maternal outcomes in women with confirmed preeclampsia. sFlt‐1 and the sFlt‐1/PlGF ratio may have a modest predictive performance for individual adverse maternal events such as thrombocytopenia, elevated liver enzymes, and acute kidney injury. Results remained consistent across subgroup analyses of early‐ and late‐onset, as well as preterm and term preeclampsia. Excluding postpartum hemorrhage from the composite outcome slightly improved the predictive performance of sFlt1, but did not materially alter the overall finding of poor to, at best, moderate predictive ability.

Before our study, two systematic reviews published in 2018 and 2024 reported on studies using angiogenic markers as predictors of adverse maternal outcomes in hypertensive disorders of pregnancy and concluded that there is insufficient evidence to support their use in clinical practice [3, 4]. Most studies were conducted in populations with suspected preeclampsia. The included outcomes differed between the studies, and none used the core‐outcome set that we used in our study. Six of the studies included participants with an established diagnosis of preeclampsia and thus can be compared with our results. Of these six studies, four reported moderate‐to‐good performance of angiogenic markers for different adverse maternal outcomes in preeclampsia. One study found that sFlt‐1 had a moderate predictive performance, while the others tested the sFlt‐1/PlGF ratio at different cutoffs and different outcome variants [8, 9, 10, 11]. The last two of the six comparable studies reported results similar to those of our study, with a poor‐to‐moderate predictive accuracy for the PlGF/sFlt‐1 or the sFlt‐1/PlGF ratio [12, 13]. Pooling the results from the above studies is challenging. Although certain tests perform well, most tests have exhibited poor‐to‐moderate performance, in agreement with our results [11, 14, 15, 16].

In slightly different populations—such as women hospitalized with a hypertensive diagnosis of pregnancy or women with suspected preeclampsia at triage in an obstetric referral center—and when evaluating somewhat different composite outcomes (including severe hypertension, thrombocytopenia, elevated liver enzymes, severe upper‑right quadrant or epigastric pain, progressive renal insufficiency, cerebral or visual disturbances, or persistent headache), previous studies have reported good‐to‐excellent predictive performance [11, 17]. It is not known whether this discrimination is due to identifying preeclampsia or predicting complications of preeclampsia.

To be able to compare predictive accuracy across studies, it is essential to use a similar study design in terms of the population, cutoff values for predictors, platform for analyses, and core outcomes. In addition, validation cohorts are essential to avoid overfitting. The best performance in our study was found when testing sFlt‐1 for the individual outcomes of thrombocytopenia, elevated liver enzymes, and acute kidney injury. This finding does not align with those of previous studies and needs to be validated in other larger, well‐defined cohorts from high‐income countries, using the same core outcome set. Future studies should also focus on low‐ and middle‐income countries, where a higher prevalence of severe maternal outcomes may enable analyses of adverse maternal events with few events in our cohort.

For predicting a diagnosis of preeclampsia, angiogenic markers have only proven useful in women with suspected preeclampsia before 37 gestational weeks [11, 15]. In addition, an abnormal sFlt‐1/PlGF ratio is more common in preterm than term preeclampsia [12]. Therefore, we performed four subgroup analyses to investigate the predictive performance of angiogenic markers for the composite and individual outcomes in early‐ and late‐onset and in preterm and term disease. The predictive performance remained similar. Thus, in our setting, these markers do not seem useful in women with a confirmed diagnosis of preeclampsia, even when occurring in early‐onset or preterm preeclampsia. While our findings are in line with earlier work indicating limited clinical utility of angiogenic markers in women with confirmed preeclampsia, it is important to note that other investigations have reported strong predictive performance, underscoring the heterogeneity of findings in this field. The use of angiogenic markers should be limited to women with suspected disease before 37 gestational weeks, for which the predictive accuracy of a preeclampsia diagnosis is high [2].

The placenta is the main source of circulating concentrations of sFlt‐1 and PlGF in pregnancy and preeclampsia. Though there are also other contributing sources to circulating concentrations, to a lesser extent, such as sequestering in the endothelial glycocalyx, PlGF is also expressed in low levels in many tissues, such as thyroid, heart, lung, skeletal muscle, and bone [18, 19, 20, 21, 22]. The lack of predictive performance of PlGF for adverse outcomes in preeclampsia could partly be explained by secretion from these other sources, confounding the decreased secretion from the placenta.

We did not investigate the performance of angiogenic biomarkers in predicting fetal and neonatal complications. Studies have shown that the sFlt‐1/PlGF ratio could be used to rule out perinatal death, neonatal ICU admission, intraventricular hemorrhage, preterm birth, and small for gestational age, as well as to rule in preterm birth, small for gestational age, neonatal ICU admission, and respiratory distress syndrome with a need for ventilatory support [10, 23]. This might be a field where angiogenic biomarkers could prove to be useful.

### 4.1 Strengths and limitations

A major strength of our study is its use of a Delphi consensus core‐outcome set, which enables future studies to confirm or refute our results. Another strength is the prospective design and well‐defined diagnosis of preeclampsia in our population.

We observed an association between adverse maternal outcomes and a lower incidence of preterm birth. This finding appears paradoxical, as early‐onset preeclampsia is typically linked to both increased disease severity and a higher risk of preterm birth. One possible explanation is that participants with preterm preeclampsia gave birth before adverse maternal events could arise, potentially reflecting effective obstetric management. However, this may also influence the observed incidence of adverse events in our cohort and may introduce a competing risk bias.

Some adverse maternal events according to the core outcomes used were rare in our population. The most frequent adverse maternal event was postpartum hemorrhage, and the angiogenic markers were poor predictors of postpartum hemorrhage. Therefore, our results might not be generalizable to settings with a higher incidence of severe preeclampsia or to settings with a higher association between postpartum hemorrhage and preeclampsia.

## 5 CONCLUSIONS

The angiogenic markers sFlt‐1 and PlGF have limited value in predicting adverse maternal events in women with a diagnosis of preeclampsia in a high‐income setting, irrespective of early or late disease. Use of these markers should be restricted to women with suspected preeclampsia before 37 gestational weeks to rule in or rule out the diagnosis.

## AUTHOR CONTRIBUTIONS

**Henrik Imberg**: Conceptualization; formal analysis; methodology; writing—review and editing; supervision. **Niclas Carlberg**: Conceptualization; investigation; funding acquisition; writing—original draft; writing—review and editing; methodology; validation; visualization; project administration; formal analysis; data curation. **Camilla Hesse**: Conceptualization; methodology; writing—review and editing; supervision. **Lina Bergman**: Conceptualization; investigation; funding acquisition; methodology; validation; visualization; writing—review and editing; formal analysis; project administration; supervision; resources; data curation. **Sven‐Egron Thörn**: Conceptualization; writing—review and editing; methodology; visualization; project administration; supervision. **Verena Sengpiel**: Conceptualization; investigation; writing—review and editing; methodology; project administration; formal analysis. **Malin Andersson**: Investigation; writing—review and editing; validation; project administration. **Nicole Wallin**: Investigation; writing—review and editing; validation; project administration; data curation. **Tor Damen**: Conceptualization; writing—review and editing; supervision.

## CONFLICT OF INTEREST STATEMENT

H.I. has served as a consultant for DiaMedica Therapeutics Inc., unrelated to this work. V.S. received drugs for a randomized controlled trial on the induction of labor from Norgine, unrelated to this work. L.B. received free reagents for sFlt‐1 and PlGF for preeclampsia projects from Thermo Fisher, Roche, and Revvity. L.B. received drugs and placebo for a randomized controlled trial in preeclampsia from Merk Damstadt, unrelated to this work. L.B. has been a consultant for DiaMedica, investigating new compounds to treat preeclampsia, unrelated to this work. All other authors declare no conflicts of interest.

## ETHICS STATEMENT

Ethical approval was obtained on December 28, 2018, from the Ethical Review Board Gothenburg (No. 955–18), with amendments approved on April 28, 2023, by Ethix, the Swedish Ethical Review Authority (No. 2023‐02‐271‐02).

## Supporting information



Supporting Information

## Data Availability

The data that support the findings of this study are available from the corresponding author upon reasonable request. All shared data will be anonymized to ensure participant confidentiality.
