# Supplementary material for: Soluble fms‐like tyrosine kinase‐1 and placental growth factor as predictors of adverse maternal events in women with a confirmed diagnosis of preeclampsia
Source: Pregnancy (Hoboken). 2026 Apr 23;2(3):e70293. doi: 10.1002/pmf2.70293 (PMC13344271; doi:10.1002/pmf2.70293)
Supplement: Supplementary file 1 [file PMF2-2-e70293-s001.docx]

**Supplementary material to “Soluble fms-like tyrosine kinase-1 and placental growth factor as predictors of adverse pregnancy-related events in women with a confirmed diagnosis of preeclampsia”**

Contents

[Supplementary Table S1	3](#_Toc224291583)

[Supplementary Table S2	4](#_Toc224291584)

[Supplementary Table S3	5](#_Toc224291585)

[Supplementary Table S4	6](#_Toc224291586)

[Supplementary Table S5	7](#_Toc224291587)

[Supplementary Table S6	10](#_Toc224291588)

[Supplementary Table S7	12](#_Toc224291589)

[Supplementary Table S9	17](#_Toc224291590)

[Supplementary Figure S1	19](#_Toc224291591)

[Supplementary Figure S2	20](#_Toc224291593)

[Supplementary Figure S3	21](#_Toc224291595)

[Supplementary Figure S4	22](#_Toc224291597)

[Supplementary Figure S5	23](#_Toc224291599)

# Supplementary Table S1

A list of all maternal outcomes suggested as “a core set of outcomes for preeclampsia research” established using a Delphi process and published by Duffy et al, and the definitions employed to classify outcomes during medical chart analysis

| Organ dysfunction/complication | Definitions | |
| --- | --- | --- |
| Maternal death | | Medical charts information on maternal death |
| Eclampsia | | Generalised tonic-clonic seizures in a woman with preeclampsia. ICD code O15 |
| Stroke | | ICD code I60, I61, I63 and I64 |
| Cortical blindness | | PRES diagnosed in medical charts OR radiological evidence of PRES OR diagnose I67.83 |
| Retinal detachment | | ICD-code H33.0 |
| Pulmonary edema | | Pulse oximetry ≤90 AND auscultatory crackles OR x-ray signs of pulmonary edema OR ultrasound signs of pulmonary edema |
| Acute kidney injury | | Creatinine ≥90 µmol/L or oliguria <500 ml/day |
| Liver capsule hematoma or rupture | | Radiological or surgical evidence of hematoma or rupture |
| Placental abruption | | Placental abruption diagnosed in medical charts OR ICD code O45 |
| Postpartum haemorrhage | | More than 500 ml bleeding if vaginal birth OR more than 1000 ml if caesarean section |
| Raised liver enzymes | | ALT or AST ≥2 times highest normal value (ALT >90 U/L and AST <72 U/L) |
| Low platelets | | Platelet particle concentration <100 x 10^9^/L |
| Admission to intensive care unit | | Admission to intensive care unit |
| Intubation and mechanical ventilation | | Intubation (not for childbirth) |

Notes: ALT, alanine aminotransferase; AST, aspartate aminotransferase; ICD, international classification of diseases; ICU, intensive care unit; PRES, posterior reversible encephalopathy syndrome

# Supplementary Table S2

Angiogenic biomarkers in relation to gestational age at blood sampling.

| **Biomarker** | **Intercept** | **Slope (std error)** | **Quadratic term (std error)** | **r^2^** | **p-value for trend** | |
| --- | --- | --- | --- | --- | --- | --- |
| sFlt-1 (pg/mL) | 12441.4 | − 30.473 (5.805) |  | 0.083 | <0.001 | |
| PlGF (pg/mL) | −124.84 | 1.031 (1.416) | −0.00116 (0.0030) | 0.063 | <0.001 | |
| sFlt-1/PlGF | 1543.8 | −7.834 (9.372) | 0.00902 (0.0197) | 0.078 | <0.001 | |
| ln(sFlt-1) | 9.778 | −0.006 (0.001) |  | 0.067 | <0.001 | |
| ln(PlGF) | −2.692 | 0.44 (0.18) | 0.0000715 (0.0000379) | 0.170 | <0.001 | |
| ln(sFlt-1/PlGF) | 9.854 | −0.028 (0.026) | 0.0000241 (0.0000550) | 0.184 | <0.001 | |
| Statistical analyses were performed using linear regression with the biomarker or log-transformed biomarker as outcome and gestational age at sampling as explanatory variable.  **Abbreviations:** std error, standard error; PlGF, placental growth factor; sFlt-1, soluble fms-like tyrosine kinase 1, pg picograms; mL, milliliters. | | | | | |  |

# Supplementary Table S3

Cut-offs based on Bremner et al.^7^ and manufacturer’s recommendations for PlGF and sFlt-1/PlGF to predict the diagnosis of preeclampsia, used here to predict any maternal adverse event in women with preeclampsia, and their corresponding sensitivities, specificities, positive and negative predictive values, positive and negative likelihood ratios.

| **Outcome/Biomarker/Criterion** | **Sensitivity** | **Specificity** | **PPV** | **NPV** | **LR+** | **LR−** |
| --- | --- | --- | --- | --- | --- | --- |
| **PlGF** |  |  |  |  |  |  |
| Rule-in <50 pg/mL all women | 0.50  (0.41–0.59) | 0.47  (0.39–0.54) | 0.40  (0.33–0.48) | 0.56  (0.48–0.64) | 0.93  (0.74–1.16) | 1.08  (0.86–1.36) |
| Rule-in <50 pg/mL <34 w | 0.69  (0.48–0.86) | 0.14  (0.05–0.29) | 0.36  (0.23–0.51) | 0.38  (0.14–0.68) | 0.80  (0.60–1.07) | 2.28  (0.84–6.18) |
| Rule-in <50 pg/mL ≥34 w | 0.45  (0.35–0.55) | 0.55  (0.47–0.64) | 0.42  (0.33–0.52) | 0.58  (0.49–0.66) | 1.00  (0.75–1.33) | 1.00  (0.80–1.26) |
| Rule-out >150 pg/mL all women | 0.97  (0.94–0.99) | 0.05  (0.02–0.11) | 0.59  (0.53–0.64) | 0.58  (0.28–0.85) | 1.03  (0.98–1.08) | 0.52  (0.17–1.59) |
| Rule-out >150 pg/mL <34 w | 1.00  (0.87–1.00) | 0.00  (0.00–0.09) | 0.41  (0.29–0.54) | NA* | 1.00 | NA* |
| Rule-out >150 pg/mL ≥34 w | 0.93  (0.86–0.97) | 0.04  (0.01–0.08) | 0.41  (0.35–0.48) | 0.42  (0.15–0.72) | 0.97  (0.91–1.03) | 1.92  (0.63–5.87) |
|  |  |  |  |  |  |  |
| **sFlt-1/PlGF** |  |  |  |  |  |  |
| Rule-in ≥70 <34 w | 0.92  (0.75–0.99) | 0.22  (0.10–0.38) | 0.45  (0.32–0.60) | 0.80  (0.44–0.97) | 1.18  (0.96–1.44) | 0.36  (0.08–1.54) |
| Rule-in ≥90 ≥34 w | 0.40  (0.30–0.50) | 0.65  (0.56–0.72) | 0.45  (0.35–0.56) | 0.59  (0.51–0.67) | 1.12  (0.81–1.55) | 0.93  (0.76–1.14) |
| Rule-out <50 all women | 0.75  (0.67–0.82) | 0.26  (0.20–0.34) | 0.43  (0.36–0.49) | 0.59  (0.48–0.70) | 1.02  (0.89–1.17) | 0.94  (0.64–1.38) |
| Rule-out <50 <34 w | 0.96  (0.80–1.00) | 0.16  (0.06–0.31) | 0.43  (0.30–0.57) | 0.86  (0.42–1.00) | 1.14  (0.97–1.34) | 0.25  (0.03–1.98) |
| Rule-out <50 ≥34 w | 0.70  (0.60–0.79) | 0.29  (0.22–0.37) | 0.42  (0.34–0.50) | 0.57  (0.45–0.69) | 0.99  (0.84–1.16) | 1.04  (0.70–1.53) |
|  |  |  |  |  |  |  |

Sensitivity, specificity, PPV, NPV, LR+ and LR− are given as value (95% confidence interval).

*No women with PlGF >150 pg/mL with GA <34 w.

**Abbreviations:** GA, gestational age; LR+, positive likelihood ratio; LR−, negative likelihood ratio; MoM, multiple of median; NPV, negative predictive value; PlGF, placental growth factor; PPV, positive predictive value; ROC, receiver operating characteristic; sFlt-1, Soluble FMS-like tyrosine kinase 1; w, weeks.

# Supplementary Table S4

Optimal gestational age-adjusted cut-offs for angiogenic biomarkers predicting adverse maternal events, and their corresponding sensitivities, specificities, positive and negative predictive values, positive and negative likelihood ratios, for women with preeclampsia with liveborn offspring.

| **Biomarker/Criterion** | **Cut-off** | **Sensitivity** | **Specificity** | **PPV** | **NPV** | **LR+** | **LR−** |
| --- | --- | --- | --- | --- | --- | --- | --- |
| **sFlt-1** |  |  |  |  |  |  |  |
| Youden’s J-index | −0.025 | 0.67  (0.58–0.75) | 0.5  (0.42–0.57) | 0.49  (0.42–0.57) | 0.68  (0.59–0.76) | 1.34  (1.10–1.62) | 0.66  (0.59–0.76) |
| Minimal distance | 0.073 | 0.59  (0.50–0.68) | 0.56  (0.48–0.63) | 0.49  (0.41–0.58) | 0.66  (0.57–0.73) | 1.35  (1.08–1.68) | 0.73  (0.57–0.93) |
|  |  |  |  |  |  |  |  |
| **PlGF** | −0.114 | 0.46  (0.37–0.55) | 0.59  (0.51–0.66) | 0.45  (0.36–0.54) | 0.60  (0.52–0.67) | 1.12  (0.86–1.45) | 0.92  (0.75–1.12) |
|  |  |  |  |  |  |  |  |
| **sFlt-1/PlGF** |  |  |  |  |  |  |  |
| Youdens J-index | 0.265 | 0.46  (0.37–0.55) | 0.67  (0.59–0.74) | 0.50  (0.41–0.59) | 0.63  (0.56–0.70) | 1.38  (1.04–1.83) | 0.81  (0.67–0.98) |
|  |  |  |  |  |  |  |  |
| Minimal distance | 0.205 | 0.48  (0.40–0.57) | 0.64  (0.56–0.71) | 0.49  (0.40–0.58) | 0.63  (0.56–0.70) | 1.34  (1.03–1.75) | 0.81  (0.66–0.99) |
|  |  |  |  |  |  |  |  |

Sensitivity, specificity, PPV, NPV, LR+ and LR− are given as number (95% confidence interval).

The outcome was any adverse maternal event as defined by the core outcome set for preeclampsia research by Duffy et al.^5^

Cut-off values are based on log-transformed, gestational age-adjusted multiples of median (log MoM).

If not specified, optimal cut-off values were equal when calculated with Youden’s J-index and Minimal distance.

Youden’s J-index: maximizing the sum of the sensitivity and specificity.

Minimal distance: minimizing the distance from the ROC curve to the optimal point (0, 1).

sFlt-1 MoM = sFlt-1 / exp(9.778 – 0.00585 *** GA).

PlGF MoM = PlGF / exp(−2.692 + 0.44 * GA – 0.0000715 * GA^2^).

sFlt-1/PlGF MoM = sFlt-1/PlGF ratio / exp(9.854 − 0.02763 * GA + 0.0000241 *** GA^2^).

**Abbreviations:** GA, gestational age; LR+, positive likelihood ratio; LR−, negative likelihood ratio; MoM, multiple of median; NPV, negative predictive value; PlGF, placental growth factor; PPV, positive predictive value; ROC, receiver operating characteristic; sFlt-1, Soluble FMS-like tyrosine kinase 1.

# Supplementary Table S5

Optimal gestational age-adjusted cut-offs for angiogenic biomarkers predicting individual adverse maternal events, and their corresponding sensitivities, specificities, positive and negative predictive values, positive and negative likelihood ratios, in women with preeclampsia.

| **Outcome/Biomarker/Criterion** | **Cut-off** | **Sensitivity** | **Specificity** | **PPV** | **NPV** | **LR+** | **LR−** |
| --- | --- | --- | --- | --- | --- | --- | --- |
| **Thrombocytopenia** |  |  |  |  |  |  |  |
| **sFlt-1** | 0.261 | 0.72  (0.47–0.90) | 0.69  (0.63–0.74) | 0.13  (0.07–0.21) | 0.98  (0.94–0.99) | 2.32  (1.66–3.24) | 0.40  (0.19–0.85) |
|  |  |  |  |  |  |  |  |
| **PlGF** |  |  |  |  |  |  |  |
| Youden J-index | −0.308 | 0.44  (0.22–0.69) | 0.71  (0.65–0.76) | 0.09  (0.04–0.16) | 0.95  (0.92–0.98) | 1.53  (0.88–2.64) | 0.78  (0.51–1.19) |
| Minimal distance | −0.207 | 0.50  (0.26–0.74) | 0.64  (0.58–0.70) | 0.08  (0.04–0.15) | 0.95  (0.91–0.98) | 1.39  (0.85–2.26) | 0.78  (0.49–1.25) |
|  |  |  |  |  |  |  |  |
| **sFlt-1/PlGF** |  |  |  |  |  |  |  |
| Youden J-index | 0.651 | 0.50  (0.26–0.74) | 0.81  (0.76–0.85) | 0.14  (0.07–0.25) | 0.96  (0.93–0.98) | 2.63  (1.56–4.42) | 0.62  (0.39–0.98) |
| Min distance | 0.332 | 0.61  (0.36–0.83) | 0.66  (0.60–0.71) | 0.10  (0.05–0.17) | 0.96  (0.93–0.99) | 1.78  (1.19–2.67) | 0.59  (0.33–1.06) |
|  |  |  |  |  |  |  |  |
| **Elevated liver enzymes** |  |  |  |  |  |  |  |
| **sFlt-1** | 0.163 | 0.65  (0.46–0.80) | 0.64  (0.58–0.69) | 0.18  (0.12–0.26) | 0.94  (0.89–0.97) | 1.78  (1.33–2.39) | 0.55  (0.35–0.88) |
|  |  |  |  |  |  |  |  |
| **PlGF** |  |  |  |  |  |  |  |
| Youden J-index | −1.462 | 0.03  (0.00–0.15) | 0.99  (0.97–1.00) | 0.33  (0.01–0.91) | 0.89  (0.85–0.92) | 4.01  (0.37–43.11) | 0.98  (0.92–1.04) |
| Min distance | −0.223 | 0.26  (0.13–0.44) | 0.63  (0.57–0.69) | 0.08  (0.04–0.15) | 0.87  (0.82–0.92) | 0.72  (0.40–1.29) | 1.16  (0.93–1.45) |
|  |  |  |  |  |  |  |  |
| **sFlt-1/PlGF** | 0.312 | 0.47  (0.30–0.65) | 0.64  (0.58–0.70) | 0.14  (0.08–0.22) | 0.91  (0.86–0.94) | 1.32  (0.90–1.96) | 0.82  (0.59–1.14) |
|  |  |  |  |  |  |  |  |
| **Acute kidney injury** |  |  |  |  |  |  |  |
| **sFlt-1** |  |  |  |  |  |  |  |
| Youden J-index | 0.413 | 0.54  (0.33–0.74) | 0.82  (0.77–0.86) | 0.20  (0.11­–0.32) | 0.95  (0.92–0.98) | 2.95  (1.89–4.59) | 0.56  (0.36–0.87) |
| Min distance | 0.311 | 0.58  (0.37–0.78) | 0.74  (0.69–0.79) | 0.16  (0.09–0.26) | 0.95  (0.92–0.98) | 2.26  (1.53–3.35) | 0.56  (0.35–0.91) |
|  |  |  |  |  |  |  |  |
| **PlGF** | −0.134 | 0.50  (0.29–0.71) | 0.59  (0.53–0.64) | 0.09  (0.05–0.16) | 0.93  (0.89–0.96) | 1.21  (0.79–1.85) | 0.85  (0.56–1.29) |
|  |  |  |  |  |  |  |  |
| **sFlt-1/PlGF** |  |  |  |  |  |  |  |
| Youden J-index | 0.967 | 0.42  (0.22–0.63) | 0.91  (0.87–0.94) | 0.28  (0.14–0.45) | 0.95  (0.91–0.97) | 4.54  (2.49–8.25) | 0.64  (0.46–0.90) |
| Min distance | 0.612 | 0.50  (0.29–0.71) | 0.80  (0.75–0.85) | 0.18  (0.09–0.29) | 0.95  (0.91–0.97) | 2.53  (1.59–4.02) | 0.62  (0.42–0.93) |
|  |  |  |  |  |  |  |  |
| **Postpartum hemorrhage** |  |  |  |  |  |  |  |
| **sFlt-1** | −0.025 | 0.65  (0.53–0.75) | 0.46  (0.39–0.53) | 0.30  (0.24–0.38) | 0.78  (0.70–0.85) | 1.19  (0.98–1.46) | 0.77  (0.56–1.07) |
|  |  |  |  |  |  |  |  |
| **PlGF** | −0.055 | 0.56  (0.45–0.67) | 0.55  (0.48–0.62) | 0.31  (0.24–0.39) | 0.78  (0.70–0.84) | 1.25  (0.98–1.59) | 0.80  (0.61–1.05) |
|  |  |  |  |  |  |  |  |
| **sFlt-1/PlGF** | 0.073 | 057  (0.46–0.68) | 0.55  (0.48–0.61) | 0.32  (0.24–0.40) | 0.78  (0.71–0.84) | 1.26  (1.00–1.60) | 0.78  (0.59–1.03) |
|  |  |  |  |  |  |  |  |

Sensitivity, specificity, PPV, NPV, LR+ and LR− are given as value (95% confidence interval).

Cut-off values are based on log-transformed, gestational age-adjusted multiples of median (log MoM).

If not specified, optimal cut-off values were equal when calculated with Youden’s J-index and Minimal distance.

Youden’s J-index: maximising the sum of the sensitivity and specificity.

Minimal distance: minimising the distance from the ROC curve to the optimal point (0, 1).

sFlt-1 MoM = sFlt-1 / exp(9.778 – 0.00585 *** GA).

PlGF MoM = PlGF / exp(−2.692 + 0.44 * GA – 0.0000715 * GA^2^).

sFlt-1/PlGF MoM = sFlt-1/PlGF ratio / exp(9.854 − 0.02763 * GA + 0.0000241 *** GA^2^).

**Abbreviations:** GA, gestational age; LR+, positive likelihood ratio; LR−, negative likelihood ratio; MoM, multiple of median; NPV, negative predictive value; PlGF, placental growth factor; PPV, positive predictive value; ROC, receiver operating characteristic; sFlt-1, Soluble FMS-like tyrosine kinase 1.

# Supplementary Table S6

Maternal and fetal characteristics at enrollment and delivery in women with preeclampsia, stratified by early on-set or late on-set and preterm or term preeclampsia.

| Maternal or fetal characteristics | Early-onset | Late-onset | Preterm | Term |
| --- | --- | --- | --- | --- |
|  | n=63 | n=244 | n=128 | n=179 |
|  |  |  |  |  |
| Age (years) | 32 (5.8) | 31 (4.7) | 32 (5.5) | 31 (4.5) |
| Nulliparous | 43 (68%) | 196 (80%) | 95 (74%) | 144 (80%) |
| Multipara | ≤3 (≤4.8%) | 4 (1.6%) | 4 (3.1%) | ≤3 (≤1.7%) |
| Body mass index (kg/m^2^) | 27.4 (5.9) | 26.6 (5.6) | 27.1 (5.5) | 26.5 (5.8) |
| Diabetes mellitus type I | ≤3 (≤4.8%) | ≤3 (≤1.2%) | ≤3 (≤2.3%) | ≤3 (≤1.7%) |
| Diabetes mellitus type II | ≤3 (≤4.8%) | ≤3 (≤1.2%) | ≤3 (≤2.3%) | ≤3 (≤1.7%) |
| Gestational diabetes | ≤3 (≤4.8%) | 9 (3.7%) | 5 (3.9%) | 6 (3.4%) |
| Chronic hypertension | 6 (9.5%) | 8 (3.3%) | 10 (7.8%) | 4 (2.2%) |
| Renal disease | ≤3 (≤4.8%) | ≤3 (≤1.2%) | ≤3 (≤2.3%) | ≤3 (≤1.7%) |
| Smoking during pregnancy | ≤3 (≤4.8%) | 11 (4.5%) | 4 (3.1%) | 8 (4.5%) |
| Alcohol use during pregnancy | ≤3 (≤4.8%) | ≤3 (≤1.2%) | ≤3 (≤2.3%) | ≤3 (≤1.7%) |
| **At inclusion** |  |  |  |  |
| Gestational age at blood sampling (weeks+days) | 31+1  (28+2–32+3) | 37+6  (36+4–39+3) | 34+0  (31+1–35+3) | 38+4  (37+5–39+6) |
| Systolic blood pressure (mmHg) | 148 (15) | 142 (13) | 147 (15) | 141 (12) |
| Diastolic blood pressure (mmHg) | 92 (10) | 89 (9) | 92 (10) | 88 (8) |
| Hemoglobin (g/dL) | 12.1 (1.2) | 12.0 (1.0) | 11.9 (1.1) | 12.1 (1.0) |
| Platelet count (×10^9^) | 223 (61) | 208 (56) | 216 (55) | 208 (59) |
| Creatinine (µmol/L) | 58 (13) | 56 (11) | 57 (12) | 56 (11) |
| Alanine aminotransferase (U/L) | 22.8 (16.2) | 16.2 (12.6) | 19.8 (15.0) | 15.6 (12) |
| Duration of antihypertensive treatment before delivery (days) | 11 (5–29) | 3 (1–10) | 10 (5–24) | 2 (1–5) |
| **At delivery** |  |  |  |  |
| Gestation at delivery  (weeks+days) | 32+2  (28+6–34+0) | 38+1 (37+1−39+4) | 35+0 (32+2−36+2) | 38+6 (37+6−40+0) |
| Days from diagnosis to delivery | 9 (4–19) | 3 (2–6) | 8 (5–14) | 3 (2–4) |
| Liveborn | 61 (97%) | 244 (100%) | 126 (98%) | 179 (100%) |
| Birthweight (g) | 1404 (511) | 3116 (643) | 1984 (782) | 3323 (541) |
| Birthweight centil | 5.8 (10.8) | 41.2 (32.0) | 17.3 (26.1) | 45.9 (31.0) |
| Vaginal delivery | 7 (11%) | 160 (66%) | 92 (72%) | 131 (73%) |
| Cesarean section | 56 (89%) | 84 (34%) | 36 (28%) | 48 (27%) |
| Severe hypertension | 58 (92.1%) | 156 (63.9%) | 111 (86.7%) | 103 (57.5%) |

Numeric variables are presented as mean (standard deviation), or median (interquartile range) as appropriate. Categorical variables are presented as counts and percentages. Severe hypertension is defined as blood pressure >160/110.

In the early-onset group, one patient is missing data on platelet count. In the late-onset group, one patient is missing data on blood pressure at inclusion, two patients are missing data on creatinine, and one patient is missing data on alanine aminotransferase. In the preterm group, one patient is missing data on blood pressure, one patient is missing data on platelet count and one patient is missing data on alanine aminotransferase. In the term group two patients are missing data on creatinine.

# Supplementary Table S7

Optimal gestational age-adjusted cut-offs for angiogenic biomarkers predicting the composite outcome without postpartum hemorrhage, and their corresponding sensitivities, specificities, positive and negative predictive values, positive and negative likelihood ratios, in women with preeclampsia.

| **Outcome/Biomarker/Criterion** | **Cut-off** | **Sensitivity** | **Specificity** | **PPV** | **NPV** | **LR+** | **LR−** |
| --- | --- | --- | --- | --- | --- | --- | --- |
| **All women** |  |  |  |  |  |  |  |
| **sFlt-1** |  |  |  |  |  |  |  |
| Youden J-index | 0.229 | 0.59  (0.46–0.71) | 0.71 (0.65–0.77) | 0.36 (0.27–0.46) | 0.86 (0.81–0.91) | 2.06 (1.56–2.74) | 0.57 (0.42–0.77) |
| Minimal distance | 0.158 | 0.64 (0.51–0.75) | 0.66 (0.60–0.72) | 0.34 (0.26–0.43) | 0.87 (0.81–0.91) | 1.87 (1.45–2.41) | 0.55 (0.40–0.77) |
|  |  |  |  |  |  |  |  |
| **PlGF** |  |  |  |  |  |  |  |
| Youden J-index | −0.308 | 0.33  (0.22–0.46) | 0.71  (0.65–0.77) | 0.24  (0.16–0.34) | 0.80  (0.74–0.85) | 1.15  (0.77–1.70) | 0.94  (0.78–1.13) |
| Minimal distance | −0.134 | 0.44 (0.32–0.57) | 0.59 (0.52–0.65) | 0.22 (0.16–0.31) | 0.79 (0.73–0.85) | 1.06 (0.78–1.45) | 0.96 (0.75–1.22) |
|  |  |  |  |  |  |  |  |
| **sFlt-1/PlGF** |  |  |  |  |  |  |  |
| Youden J-index | 0.536 | 0.41 (0.29–0.54) | 0.78 (0.72–0.83) | 0.34 (0.24–0.45) | 0.83 (0.77–0.87) | 1.86 (1.28–2.71) | 0.76 (0.61–0.94) |
| Min distance | 0.276 | 0.52 (0.39–0.64) | 0.66 (0.60–0.72) | 0.29 (0.21–0.38) | 0.83 (0.77–0.88) | 1.51 (1.13–2.03) | 0.73 0.56–0.96) |
|  |  |  |  |  |  |  |  |
| **Early-onset preeclampsia** |  |  |  |  |  |  |  |
| **sFlt-1** | 0.241 | 0.59  (0.36–0.79) | 0.78  (0.62–0.89) | 0.59  (0.36–0.79) | 0.78  (0.62–0.89) | 2.69  (1.37–5.28) | 0.52  (0.31–0.89) |
|  |  |  |  |  |  |  |  |
| **PlGF** | 0.306 | 0.41  (0.21–0.64) | 0.68  (0.52–0.82) | 0.41  (0.21–0.64) | 0.68  (0.52–0.82) | 1.29  (0.66–2.53) | 0.87  (0.58–1.30) |
|  |  |  |  |  |  |  |  |
| **sFlt-1/PlGF** |  |  |  |  |  |  |  |
| Youden J-index | 0.961 | 0.32  (0.14–0.55) | 0.90  (0.77–0.97) | 0.64  (0.31–0.89) | 0.71  (0.57–0.83) | 3.26  (1.07–9.94) | 0.76  (0.56–1.02) |
| Minimal distance | 0.272 | 0.55  (0.32–0.76) | 0.63  (0.47–0.78) | 0.44  (0.25–0.65) | 0.72  (0.55–0.86) | 1.49  (0.86–2.60) | 0.72  (0.43–1.20) |
|  |  |  |  |  |  |  |  |
| **Late-onset preeclampsia** |  |  |  |  |  |  |  |
| **sFlt-1** |  |  |  |  |  |  |  |
| Youden J-index | −0.204 | 0.93  (0.81–0.99) | 0.39  (0.32–0.46) | 0.25  (0.19­–0.33) | 0.96  (0.90–0.99) | 1.53  (1.33–1.75) | 0.17  (0.06–0.53) |
| Minimal distance | 0.145 | 0.66  (0.50–0.80) | 0.63  (0.56–0.70) | 0.28  (0.20–0.38) | 0.89  (0.83–0.94) | 1.78  (1.35–2.35) | 0.54  (0.35–0.83) |
|  |  |  |  |  |  |  |  |
| **PlGF** |  |  |  |  |  |  |  |
| Youden J-index | −0.909 | 0.07  (0.01–0.19) | 0.96  (0.92–0.98) | 0.27  (0.06–0.61) | 0.82  (0.77–0.87) | 1.70  (0.47–6.17) | 0.97  (0.89–1.06) |
| Minimal distance | 0.134 | 0.41  (0.26–0.57) | 0.59  (0.52–0.66) | 0.18  (0.11–0.27) | 0.82  (0.75–0.88) | 1.01  (0.68–1.50) | 0.99  (0.76–1.30) |
|  |  |  |  |  |  |  |  |
| **sFlt-1/PlGF** |  |  |  |  |  |  |  |
| Youden J-index | 0.536 | 0.43  (0.28–0.59) | 0.79  (0.72–0.84) | 0.31  (0.20–0.44) | 0.86  (0.80–0.91) | 2.01  (1.31–3.09) | 0.72  (0.55–0.95) |
| Min distance | 0.312 | 0.50  (0.35–0.65) | 0.68  (0.61–0.74) | 0.25  (0.17–0.36) | 0.86  (0.80–0.91) | 1.54  (1.08–2.20) | 0.74  (0.54–1.01) |
|  |  |  |  |  |  |  |  |
| **Preterm preeclampsia** |  |  |  |  |  |  |  |
| **sFlt-1** | 0.157 | 0.58  (0.41–0.74) | 0.66  (0.56–0.76) | 0.40  (0.27–0.55) | 0.80  (0.70–0.89) | 1.73  (1.16–2.58) | 0.63  (0.42–0.95) |
|  |  |  |  |  |  |  |  |
| **PlGF** |  |  |  |  |  |  |  |
| Youdens J-index | −0.688 | 0.17  (0.06–0.33) | 0.90  (0.82–0.95) | 0.40  (0.16–0.68) | 0.73  (0.64–0.81) | 1.70  (0.65–4.44) | 0.92  (0.79–1.08) |
| Minimal distance | −0.131 | 0.44  (0.28–0.62) | 0.60  (0.49–0.70) | 0.30  (0.18–0.44) | 0.73  (0.62–0.83) | 1.11  (0.71–1.72) | 0.93  (0.66–1.30) |
|  |  |  |  |  |  |  |  |
| **sFlt-1/PlGF** |  |  |  |  |  |  |  |
| Youdens J-index | 0.961 | 0.28  (0.14–0.45) | 0.92  (0.85–0.97) | 0.59  (0.33–0.82) | 0.77  (0.68–0.84) | 3.65  (1.51–8.85) | 0.78  (0.63–0.97) |
| Minimal distance | 0.206 | 0.47  (0.30–0.65) | 0.66  (0.56–0.76) | 0.35  (0.22–0.51) | 0.76  (0.65–0.85) | 1.40  (0.89–2.20) | 0.80  (0.57–1.12) |
|  |  |  |  |  |  |  |  |
| **Term preeclampsia** |  |  |  |  |  |  |  |
| **sFlt-1** | 0.236 | 0.70  (0.51–0.85) | 0.72  (0.65–0.79) | 0.34  (0.22–0.47) | 0.92  (0.86–0.96) | 2.54  (1.79–3.61) | 0.41  (0.24–0.72) |
| **PlGF** | −0.205 | 0.43  (0.25–0.63) | 0.64  (0.56–0.72) | 0.20  (0.11–0.31) | 0.85  (0.77–0.91) | 1.22  (0.77–1.94) | 0.88  (0.63–1.23) |
| **sFlt-1/PlGF** |  |  |  |  |  |  |  |
| Youdens J-index | 0.519 | 0.50  (0.31–0.69) | 0.76  (0.68–0.82) | 0.29  (0.17–0.44) | 0.88  (0.81–0.93) | 2.07  (1.31–3.27) | 0.66  (0.46–0.95) |
| Minimal distance | 0.312 | 0.60  (0.41–0.77) | 0.65  (0.57–0.73) | 0.26  (0.16–0.38) | 0.89  (0.82–0.94) | 1.72  (1.19–2.48) | 0.61  (0.39–0.97) |
|  |  |  |  |  |  |  |  |

Sensitivity, specificity, PPV, NPV, LR+ and LR− are given as value (95% confidence interval).

Cut-off values are based on log-transformed, gestational age-adjusted multiples of median (log MoM).

If not specified, optimal cut-off values were equal when calculated with Youden’s J-index and Minimal distance.

Youden’s J-index: maximising the sum of the sensitivity and specificity.

Minimal distance: minimising the distance from the ROC curve to the optimal point (0, 1).

sFlt-1 MoM = sFlt-1 / exp(9.778 – 0.00585 *** GA).

PlGF MoM = PlGF / exp(−2.692 + 0.44 * GA – 0.0000715 * GA^2^).

sFlt-1/PlGF MoM = sFlt-1/PlGF ratio / exp(9.854 − 0.02763 * GA + 0.0000241 *** GA^2^).

**Abbreviations:** GA, gestational age; LR+, positive likelihood ratio; LR−, negative likelihood ratio; MoM, multiple of median; NPV, negative predictive value; PlGF, placental growth factor; PPV, positive predictive value; ROC, receiver operating characteristic; sFlt-1, Soluble FMS-like tyrosine kinase 1.

## **Table S8.** Counts and proportions of adverse maternal events, including the proportion occurring postpartum, in all women and in subgroups defined by early-onset, late-onset, preterm and term preeclampsia.

|  | **All women** | | **Early-onset** | | **Late-onset** | | **Preterm** | | **Term** | |
| --- | --- | --- | --- | --- | --- | --- | --- | --- | --- | --- |
| **Maternal adverse event** | **Total/postpartum** | | **Total/postpartum** | | **Total/postpartum** | | **Total/postpartum** | | **Total/postpartum** | |
| The composite outcome | 129 (42%) | 80 (62.0%) | 26 (41.3%) | 11 (42.3%) | 103 (42.2%) | 69 (67%) | 46 (35.9%) | 21 (45,7%) | 83 (46.4%) | 59 (71,1%) |
| The composite outcome without postpartum hemorrhage | 66 (21.5%) | 25 (37.9%) | 22 (34,9%) | 6 (27.3%) | 44 (18.0%) | 19 (43.2%) | 26  (20,3%) | 10 (38,5%) | 30 (16,8%) | 15 (50%) |
| Postpartum hemorrhage | 82 (26.7%) | 82 (100%) | 4 (6.3%) | 4 (100%) | 78 (32.0%) | 78 (100%) | 12 (9.4%) | 12 (100%) | 70 (39.1%) | 70 (100%) |
| Elevated liver enzymes | 34 (11.1%) | 19 (55.9%) | 10 (15.9%) | 5 (50%) | 24 (9.8%) | 14 (58.3%) | 19 (14.8%) | 8 (42,1%) | 15 (8.4%) | 11 (73,3) |
| Acute kidney injury | 24 (7.8%) | 9 (37.5%) | 7 (11.1%) | 2 (28.6%) | 17 (7.0%) | 7 (41.2%) | 14 (10.9%) | 5 (35,7%) | 10 (5.6%) | 4 (40%) |
| Low platelet count | 18 (5.9%) | 10 (55.6%) | 7 (11.1%) | 4 (57.1%) | 11 (4.5%) | 6 (54.5%) | 9 (7.0%) | 5 (55,6%) | 9 (5.0%) | 5 (55,6%) |
| Placental abruption | 5 (1.6%) | 0 (0%) | 2 (3.2%) | 0 (0%) | 3 (1.2%) | 0 (0%) | 3 (2,3%) | 0 (0%) | 2 (1,1%) | 0 (0) |

Data are presented as counts with corresponding percentages. For the “Total” columns, percentages were calculated using the number of women within each subgroup as the denominator. For the “Postpartum” columns, percentages were calculated using the total number of events in that subgroup (i.e., the value in the adjacent “Total” column as the denominator.

# Supplementary Table S9

Maternal risk (incidence rate) for the composite outcome and single outcomes, in all women and women stratified by early on-set or late on-set and preterm or term preeclampsia.

|  | All women | Early-onset | Late-onset | Preterm | Term |
| --- | --- | --- | --- | --- | --- |
|  | n = 307 | n=63 | n=244 | n=128 | n=179 |
|  |  |  |  |  |  |
| *The composite outcome* | 129 | 26 | 103 | 46 | 83 |
| Risk(events per day) | 1,50% | 1.24% | 1.59% | 1.06% | 2.00% |
| *The composite outcome without postpartum hemorrhage* | 66 | 22 | 44 | 36 | 30 |
| Risk(events per day) | 0,59% | 1,08% | 0,49% | 0,76% | 0,46% |
| *Thrombocytopenia* | 18 | 7 | 11 | 9 | 9 |
| Risk(events per day) | 0,14% | 0.24% | 0.11% | 0.15% | 0.12% |
| *Elevated liver enzymes* | 34 | 10 | 24 | 19 | 15 |
| Risk(events per day) | 0,27% | 0.36% | 0.24% | 0.35% | 0.21% |
| *Acute kidney injury* | 24 | 7 | 17 | 14 | 10 |
| Risk(events per day) | 0,18% | 0.24% | 0.17% | 0.25% | 0.14% |
| *Postpartum hemorrhage* | 82 | 4 | 78 | 12 | 70 |
| Risk(events per day) | 0,78% | 0.13% | 1.1% | 0.21% | 1.46% |

Numrical variables are presented as counts or percentages as appropriate.

# Supplementary Figure S1

# Forest plots of AUC testing sFlt-1, PlGF and sFlt-1/PlGF against composite and single outcomes for women with early-onset preeclampsia (≤33+6). Results for biomarkers are unadjusted for gestational age.

# Supplementary Figure S2

# Forest plots of AUC testing sFlt-1, PlGF and sFlt-1/PlGF against composite and single outcomes for women with late-onset preeclampsia (>33+6). Results for biomarkers are unadjusted for gestational age.

# Supplementary Figure S3

# Forest plots of AUC testing sFlt-1, PlGF and sFlt-1/PlGF against composite and single outcomes for women with preterm preeclampsia (≤36+6). Results for biomarkers are unadjusted for gestational age.

# Supplementary Figure S4

# Forest plots of AUC testing sFlt-1, PlGF and sFlt-1/PlGF against composite and single outcomes for women with preeclampsia at term (>36+6). Results for biomarkers are unadjusted for gestational age.

# Supplementary Figure S5

# Forest plots of AUC testing sFlt-1, PlGF and sFlt-1/PlGF against the composite outcome without postpartum hemorrhage for all women, and women with early-onset, late-onset, preterm, and term preeclampsia.
